# Effect of Zinc on Efficacy of Iron Supplementation in Improving Iron and Zinc Status in Women

**DOI:** 10.1155/2012/216179

**Published:** 2012-06-07

**Authors:** Phuong Nguyen, Ruben Grajeda, Paul Melgar, Jessica Marcinkevage, Rafael Flores, Usha Ramakrishnan, Reynaldo Martorell

**Affiliations:** ^1^Nutrition and Health Sciences Program, Rollins School of Public Health, Emory University, Atlanta, GA 30322, USA; ^2^Micronutrient Program, Pan American Health Organization, Washington, DC 20037, USA; ^3^Institute of Nutrition of Central America and Panama, Calzada Roosevelt 6-25 Zona 11, Apartado Postal 1188, Guatemala City, Guatemala; ^4^Hubert Department of Global Health, Rollins School of Public Health, Emory University, Atlanta, GA 30322, USA

## Abstract

Iron and zinc may interact in micronutrient supplements and thereby decrease efficacy. We investigated interactive effects of combined zinc and iron supplementation in a randomized controlled trial conducted in 459 Guatemalan women. Four groups were supplemented for 12 weeks: (1) weekly iron and folic acid (IFA); (2) weekly IFA and 30 mg zinc; (3) daily IFA; (4) daily IFA and 15 mg zinc. Effects were assessed by generalized linear regression. Baseline hemoglobin (Hb) concentration was 137.4 ± 15.5 g/L, 13% were anemic and 54% had zinc deficiency. Hb cconcentrations were similar by supplement type, but Hb concentrations improved significantly in anemic women at baseline (increase of 21.8 g/L). Mean percentage changes in serum ferritin were significantly higher in daily compared to weekly supplemented groups (86% versus 32%). The addition of zinc to IFA supplements had no significant impact on iron or zinc status. In conclusion, adding zinc to IFA supplements did not modify efficacy on iron status or improve zinc status, but daily supplementation was more efficacious than weekly in improving iron stores.

## 1. Introduction

Women of reproductive age (WRA) in poor countries are at high risk for micronutrient deficiencies, particularly iron and zinc. WHO estimates that 30.2% of nonpregnant and 41.8% of pregnant women suffer from anemia, much of it due to iron deficiency [1–3]. Information on zinc deficiency in WRA is limited. Some 1.2 billion people worldwide are at risk of inadequate zinc intake and presumably many are zinc deficient [4]. Both iron and zinc deficiencies have adverse consequences for human health. Iron deficiency results in anemia, impaired psychomotor development, reduced physical and work capacity, impaired immunity, and adverse pregnancy outcomes [5]. Zinc deficiency is associated with fertility reduction [6], poor pregnancy outcomes [7], mental and behavioral changes [8], impaired immunity, increased morbidity and mortality [9], and perhaps linear growth retardation [10, 11].

Several strategies have been implemented to address iron and zinc deficiency, including supplementation and food fortification. One approach is through combined zinc and iron supplementation. However, there is concern about potential interactions between these two trace minerals. Although some pathways are unique, iron and zinc have many similar absorption and transport mechanisms and may therefore compete for absorption [12, 13]. Several studies examined the efficacy of supplementation with iron and zinc, but most were conducted in children and involved only daily doses. Little is known regarding the influence of zinc on the efficacy of iron supplementation on a weekly basis, particularly in WRA. One study, in Bangladeshi infants [14], examined weekly supplementation of zinc, iron, and of combined iron and zinc. Results showed that weekly provision of both iron and zinc supplementation did not modify the effect of each nutrient when given alone. Recently, a meta-analysis examined the impact of zinc supplementation on biochemical indicators of iron and zinc [10]. This paper, however, did not quantify the effects of adding zinc to iron compared to iron alone on iron and zinc status. The objective of this study is to investigate the efficacy of IFA supplements provided daily or weekly with and without zinc on iron and zinc status in a randomized control trial (RCT) carried out in Guatemalan WRA.

## 2. Methods

The study design, sample size calculation, data collection, and characteristics of the Guatemalan RCT have been described in detail elsewhere [15]. Briefly, 459 nonpregnant, nonlactating women aged 15–49 years from the village of Concepción Chiquirichapa located in the western highlands of Guatemala were recruited. These women were randomly assigned to receive one of four supplements: (1) weekly 120 mg iron with 30 mg zinc, (2) weekly 120 mg iron, (3) daily 60 mg iron with 15 mg zinc, and (4) daily 60 mg iron. Iron and zinc were provided as ferrous sulphate and zinc sulphate, respectively. All supplements also contained folic acid (FA) (the weekly arms had either 5000 or 2800 *μ*g and the daily arms 400 or 200 *μ*g, resp.) and vitamin B-12 (16.8 *μ*g for the weekly arms or 2.4 *μ*g for the daily arms). Trained field workers from the community visited each woman 7 days a week to deliver and observe the ingestion of the supplements (two-three hours after a meal) for the entire 12-week duration of supplementation. All women received 7 pills per week. The weekly dose groups received 6 placebos and 1 active pill on the third day of the week. Daily records were kept to track the participants' health and compliance. The trial was registered in the US NIH Clinical Trial Registry (identification number NCT003994862).

 Dietary intake data at baseline were collected by means of a semiquantitative food frequency questionnaire; socio-demographic information was also collected at baseline [15]. The effect of supplementation on folate and homocysteine status was reported previously [15]. Here we assess the impact of supplements on iron and zinc status. Hb, serum ferritin, C-reactive protein (CRP), and serum zinc were measured pre- and post-supplementation. A capillary blood sample was obtained from a finger prick to measure Hb concentrations using a B-Hemoglobin Analyzer. Venous blood was collected for measuring serum ferritin and CRP using a Nephelometric immunoassay reactive kit and for determining serum zinc using a flame atomic absorption spectroscopy method [16]. Venous blood was collected after an overnight fast using trace mineral free syringes; tubes were centrifuged within an hour at 3,000 rpm for 10 minutes at 4°C. The serum was separated and stored at −70°C at the Institute of Nutrition of Central America and Panama (INCAP) in Guatemala City until analysis at the National Institute of Public Health (NIPH), Cuernavaca, Mexico. The time lag between the last consumption of supplement and the blood draw was similar for weekly and daily groups (2.46 ± 1.15 days versus 2.50 ± 1.02 days, *P* > 0.05).

**Figure 1:**
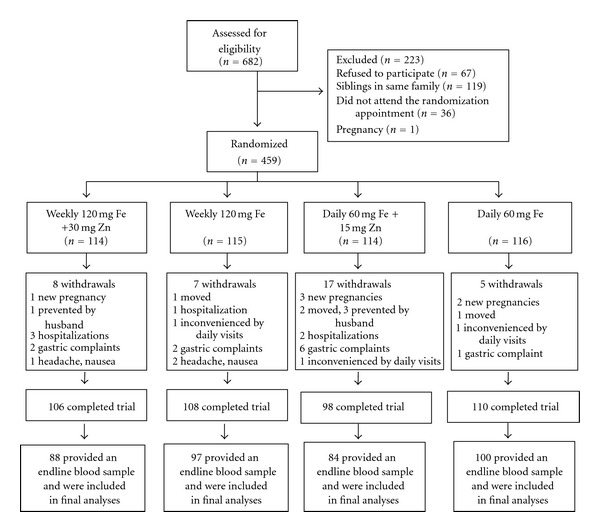
Flow chart describing participation of subjects in the study.

 Since willingness to provide a blood sample was a criterion, blood samples were available for all 459 subjects at baseline (Figure 1). Of these, 422 (92%) finished the trial; reasons for loss to followup were similar across groups, but the daily iron/zinc group had a higher dropout rate compared to the other groups (*P* = 0.02). A total of 369 women or 88% also provided an endline blood sample (i.e., 52 women refused). Analyses were performed based on these 369 subjects (88 in the weekly iron and zinc group, 97 in the weekly iron group, 84 in the daily iron and zinc group, and 100 in the daily iron group) or 80% of those randomized at baseline. Subjects included in the analyses had similar baseline characteristics compared to subjects not included (*P* > 0.05, results not shown).

Since the women lived 2600 m above sea level, Hb was adjusted for altitude using an exponential curve of Hb concentration by altitude described by Cohen and Haas [17]. Data were checked for normality using the Kolmogorov-Smirnov test of normality. Log transformation was used to normalize the distribution of serum ferritin. The effect of treatment on Hb, serum ferritin, and zinc was assessed using a generalized linear regression model (SAS Proc Mixed procedures) assuming unstructured correlation to account for the correlation among the repeated observations for a given subject [18], using treatment as a fixed factor and time as a covariate. The between-subjects factor was four treatment types and the within-subjects factor was treatment effects (from start to finish of supplementation). Between-group differences in treatment effect would be indicated by a significant interaction between treatment effect and treatment type. This is obtained by fitting the model below:

(1) $$\begin{array}{cc} \text{Outcome}\; & =\beta 0+\beta 1\times \text{time}+\beta 2\times \text{treatment}+\beta 3\\ & \;\times \text{time}\times \;\text{treatment}+Bi\times \text{other}\;\text{covariates}. \end{array}$$

The model takes baseline values into account in estimating supplement effects. We investigated further whether the effects of supplementation varied depending on initial Hb, serum ferritin, and serum zinc status. Anemia was defined as Hb value <120 g/L, insufficient iron stores were defined as serum ferritin <20 *μ*g/L, and depleted iron stores as serum ferritin <12 *μ*g/L; zinc deficiency was defined as serum zinc <10.7 *μ*mol/L.

Serum CRP concentrations were low (3.5% of values were >10 mg/L), similar at baseline and endline and across groups and their inclusion in the model (as dichotomous variables indicating >5 mg/L at baseline and endline or alternatively, >10 mg/L; or as linear and quadratic terms to account for nonlinearity) had only a trivial influence on estimates of the effect of supplementation on serum ferritin values and did not influence the zinc results (results not shown). Thus, for the sake of parsimony, we did not include CRP in the models.

All statistical tests were two-tailed, and differences were considered significant at  *P* < 0.05. SAS software, version 9.1 was used for statistical analysis [19].

## 3. Results

 There were no significant differences in selected characteristics across the four treatment groups at baseline (Table 1). Levels of Hb, serum ferritin, and zinc before and after the intervention by group are shown in Table 2. The baseline Hb concentration was 137.4 ± 15.5 g/L. Mean Hb concentrations were not significantly different across the 4 treatment groups at baseline or endline. However, the changes in Hb were strongly related to the initial Hb concentration (*r* = −0.65; *P* < 0.001). Overall Hb concentrations increased by 21.8 g/L (95% CI: 17.5, 26.2) among anemic women but decreased by 4.8 g/L (−6.5; −3.2) among nonanemic women (data not shown).

 Baseline ferritin concentrations did not differ across groups (*P* = 0.98) (Table 2). Ferritin levels increased significantly in all 4 supplementation groups after 12 weeks of supplementation (*P* < 0.001). There were differences between groups in treatment effects (*P* = 0.0006). The two groups receiving daily supplementation had significantly higher ferritin concentrations than those receiving weekly. Ferritin levels increased by 86% (95% CI: 67, 108) or 31.7 *μ*g/L in daily groups compared to 32% (95% CI: 18, 47) or 7.6 *μ*g/L in weekly groups. No significant differences were found in ferritin levels in the daily groups between those with or without zinc (*P* = 0.94) or in the weekly groups between those with or without zinc (*P* = 0.38).

 The prevalence of anemia before supplementation was 13% and that of insufficient iron stores was 19.7%. There was no difference in the proportion of anemia at baseline and endline by supplement type (Table 3). The prevalence of iron insufficiency and depletion decreased significantly but was similar across the 4 groups. Overall iron insufficiency decreased from 19.7 to 6.6% (*P* < 0.001) and iron depletion decreased from 11.5 to 2.7% (*P* < 0.001).

 The initial serum zinc concentration was 10.5 ± 2.1 *μ*mol/L. There were no significant differences across the 4 groups at baseline or endline. Serum zinc concentrations did not change in any of the intervention groups after supplementation (*P* = 0.55) (Table 2). Zinc deficiency was observed in 54.4% of women. The prevalence of zinc deficiency did not change after supplementation in any of the four groups (*P* = 0.52) (Table 3).

## 4. Discussion

 In the present study, women randomly assigned to receive daily or weekly Fe-FA supplements with or without zinc. Neither daily nor weekly supplementation changed Hb concentrations, and the addition of zinc did not influence Hb concentrations. However, Hb concentrations improved significantly in women who were anemic at baseline by an average of 21.8 g/L. For serum ferritin, significant between- and within-group changes were noted. After supplementation, daily groups showed a much greater increase in serum ferritin concentrations than the weekly groups, suggesting that daily iron supplementation was more efficacious than weekly supplementation in improving serum ferritin concentrations. Ferritin concentrations improved by 86% (or 31.7 *μ*g/L) in the daily supplement groups compared with 32% (or 7.6 *μ*g/L) in the weekly groups. The addition of zinc to supplements containing iron had no influence on serum ferritin.

Although daily or intermittent iron supplementation, with and without zinc, significantly increased serum ferritin concentration, it did not reduce the percentage of anemic women. One possible explanation is that iron deficiency may be only one of several causes of anemia. Mean Hb concentrations improved significantly in women who were anemic at baseline by 21.8 g/L, but this increase was not large enough to reduce the prevalence of anemia.

Our findings showed that adding zinc to supplements containing iron did not influence iron status but also did not improve zinc status in WRA. Therefore, adding zinc to IFA supplements may not be an optimal way to improve zinc status in women. The addition of zinc to IFA may have other benefits for women, but there is limited information. Findings from previous meta-analyses indicate that maternal zinc supplementation resulted in a 14% reduction in preterm delivery but had no significant impact on infant's birth weight nor on other pregnancy outcomes [20]. In a study in Peru [21], 1295 mothers were assigned randomly to receive prenatal supplements containing 60 mg iron and 250 *μ*g FA, with or without 15 mg zinc, beginning at 10–24 weeks of gestation. Findings from this study suggested that adding zinc to IFA tablets did not affect the duration of pregnancy, size at birth, or developmental outcomes in Peruvian children when assessed at 4.5 y of age [22] but did improve postnatal growth [23]. A study in Ghana found no difference in the mean weight of newborns of women receiving a combined supplement of zinc and iron compared to those of women receiving iron alone [24]. In a trial in Nepal [25], 4926 pregnant women were allocated at random to five regimens: daily supplements of FA, IFA, IFA with zinc, multiple micronutrients, or vitamin A alone (control). The addition of zinc to IFA reduced the efficacy of IFA on birth weight; the mean birth weight was 53 g (0 g to 108 g) lower in the IFA plus zinc group compared to the IFA group. Also, there was no difference in the proportion of preterm births between these two groups [25]. Finally, fetal loss and infant mortality did not differ between the groups who received IFA only and IFA with zinc [26, 27]. Thus, adding zinc to IFA supplements for women needs to be reconsidered since it appears to not benefit biochemical indicators or functional indicators. More studies that assess the impact of combined iron and zinc on functional status are needed to provide scientific evidence for public health policy. Ultimately, the public health benefit of adding zinc to iron supplements given to women depends on the baseline characteristics of specific populations and on the relative benefits of improving iron and zinc status.

Possible reasons for the lack of an effect of adding zinc to iron on zinc status in WRA include the negative effect of iron on the absorption of zinc. Unfortunately, the lack of a group that received zinc but no iron in the Guatemalan RCT does not allow us to test this possibility. A review by Solomons (1986) reported that competitive inhibition of zinc uptake by iron can occur at iron: zinc ratios of 2 : 1 or greater. The ratio of iron: zinc in the supplements in most studies in women is 4 : 1 while that ratio in most studies in children is 1 : 1. In addition, findings from several studies suggest that iron may have deleterious effects on zinc metabolism in women. A significant reduction in zinc absorption was reported when iron was taken with zinc in pregnant [28, 29] and lactating [30] women. In nonpregnant women, oral iron given with zinc impaired bioavailability of zinc [31, 32]. In women, iron is often provided in combination with folic acid. There is concern that folic acid supplements may impair zinc absorption [29, 33], possibly by forming an insoluble chelate in the lumen [33] or by a mutually inhibitory effect of zinc and folate on intestinal transport mechanisms [34]. However, subsequent studies failed to confirm the inhibitory effect of folate on zinc absorption [35] or on zinc status [36–38]. Another possible but unlikely explanation for the lack of effect of zinc supplementation on serum zinc levels may be that the dose of zinc used was insufficient; we used a dose that was twice the RDA [39]. Although several studies have been carried out to assess the interaction between iron and zinc, almost all of them used daily supplementation. To our knowledge, the Guatemalan study is the first RCT to investigate the influence of zinc on iron efficacy of weekly doses in women. The only other weekly dose study was conducted in Bangladeshi infants [14] who were assigned to receive weekly supplementation of 1 mg riboflavin (control), 20 mg iron, 20 mg zinc, or both for 6 months. Findings from that study were that the addition of zinc to weekly iron supplementation improved children's zinc status but had no differential effect on iron status compared to iron alone.

The main strength of our study is the RCT design; other strengths include the demonstration that randomization was effective, the direct observation of supplement intakes, and the careful standardization and high quality of measurements. While only 80% of women who were randomized at baseline were included in the analyses, those not included did not differ from those included in terms of baseline characteristics. Because of ethical concerns, women with severe anemia (Hb < 70 g/L) at baseline were treated and excluded from the trial. Thus, the true prevalence of anemia in the population is higher than the prevalence reported in the study (13%). If severely anemic women had been included in the study, the average effect of supplementation might have been larger. A limitation is the absence of a placebo group; hence, we cannot exclude the possibility that community-wide dietary improvements coincided with the beginning of supplementation. This would appear highly unlikely. Also, the lack of a zinc only group does not allow us to fully assess the interaction between iron and zinc. Another limitation of the Guatemalan RCT is that it provided nutrients other than iron and zinc. This study was designed primarily to test the relative impact of daily versus weekly folic acid (FA) supplements, with two levels of doses for each [15]. The main conclusion of the study was that weekly FA (either high or low dose) may be as efficacious as daily supplementation (either high or low dose) in improving serum folate levels. Thus, because of a similar response across all groups, we believe that the folic acid in the supplements did not influence the differential responses found across groups. The supplements also contained vitamin B-12. Both weekly groups received 16.8 *μ*g and both daily groups received 2.4 *μ*g. It was found that daily supplementation improved serum B-12 significantly while weekly supplementation had no effect. Because the key comparisons we make in our RCT are between daily supplements with iron versus iron and zinc or between weekly iron supplements with iron versus iron and zinc, vitamin B-12 is not a factor in interpreting these differences. On the other hand, B-12 would be an issue in comparisons of any weekly to any daily iron/zinc group. Finally, another possible limitation of our study is that we included only CRP which captures the effects of inflammation early in the acute phase response; it would have been appropriate to have also included alpha-1-acid glycoprotein (AGP) to capture later stages of inflammation [40]. Study women had low levels of CRP and these levels did not influence the estimation of supplement effects. CRP values were similar across groups at baseline and endline; we would expect other unmeasured, potential confounders, such as AGP, to have also been balanced across groups. For these reasons, we think it is unlikely that the omission of AGP clouds the interpretation of our results.

## 5. Conclusion

 Both daily and weekly supplementations were efficacious in improving Hb concentration in anemic women. However, daily supplementation was more efficacious than weekly in improving ferritin levels. The combined Fe-Zn supplementation was as effective as iron alone in improving iron status but not effective in improving zinc status. Other approaches must be considered to control zinc deficiency in this population.

## Figures and Tables

**Table 1:** Selected baseline characteristics of the four treatment groups.

Characteristics	Treatment groups				
	Weekly Fe + Zn (*n* = 88)	Weekly Fe (*n* = 97)	Daily Fe + Zn (*n* = 84)	Daily Fe (*n* = 100)	*P* value
Age (years)	31.0 ± 9.4^1^	31.4 ± 9.0	30.2 ± 8.9	31.6 ± 10.1	0.75^4^
Education (years)	5.5 ± 3.8	6.0 ± 4.3	6.2 ± 3.8	5.7 ± 3.5	0.66
Socioeconomic status					
Low	29 (33.3)^2^	35 (36.1)	34 (41.0)	32 (32.3)	0.38^5^
Medium	31 (35.6)	24 (24.7)	25 (30.1)	38 (38.4)	
High	27 (31.0)	38 (39.2)	24 (28.9)	29 (29.3)	
Weight (kg)	55.2 ± 9.6	54.9 ± 8.9	55.4 ± 10.7	53.5 ± 10.0	0.58
Height (cm)	145.1 ± 4.4	145.2 ± 4.7	145.1 ± 5.1	144.7 ± 4.4	0.89
Body mass index (kg/m^2^)	26.2 ± 4.3	26.1 ± 4.3	26.2 ± 4.4	25.5 ± 4.1	0.61
Compliance (%)	98.8 ± 1.9	98.9 ± 2.4	99.0 ± 1.6	99.0 ± 2.9	0.97
Dietary intake					
Folate (*μ*g)	384^3^ (292–524)	370 (252–485)	340 (246–540)	364 (281–483)	0.49^6^
Vitamin B-12 (*μ*g)	2.0 (0.7–4.1)	1.7 (0.8–3.9)	2.1 (0.7–3.2)	1.4 (0.7–3.0)	0.51
Vitamin B-6 (mg)	1.1 (0.8–1.4)	1.1 (0.7–1.5)	1.0 (0.8–1.4)	1.1 (0.7–1.4)	0.75
Iron (mg)	15.0 (11.3–21.5)	13.9 (9.7–18.1)	13.5 (10.3–17.7)	13.8 (10.7–17.5)	0.27
Zinc (mg)	9.2 (7.1–12.3)	9.2 (6.9–12.0)	9.1 (7.1–11.4)	8.6 (7.4–11.7)	0.97
Energy (kJ)	6732 (5749–8916)	6732 (5448–8477)	6661 (5594–8602)	6945 (5494–8184)	0.89

^1^Mean ± SD for age, education, height, weight, BMI, and compliance.

^2^ *n* (%) for SES.

^3^Median (interquartile range) for dietary intake.

^4^ANOVA test for age, education, height, weight, BMI, and compliance.

^5^Chi-square test for categorical variables.

^6^Kruskal-Wallis test for dietary intake.

**Table 2:** Hemoglobin, serum ferritin, and serum zinc concentrations in women before and after daily or weekly supplementation for 12 weeks (*n* = 369).

Treatment groups		Baseline	Endline	Difference
Hemoglobin, g/L	*n*	Mean^1^(95% CI)	Mean^1^ (95% CI)	Mean^3^ (95% CI)

Weekly Fe+ Zn	88	137.6 (134.3, 140.8)	135.5 (132.6, 138.4)	−2.1 (−5.8, 1.6)
Weekly Fe	97	138.7 (135.6, 141.8)	137.8 (135.0, 140.6)	−0.9 (−4.5, 2.6)
Daily Fe+ Zn	84	136.4 (133.0, 139.7)	135.3 (132.3, 138.3)	−1.0 (−4.8, 2.7)
Daily Fe	100	137.0(133.9, 140.0)	135.9 (133.2, 138.6)	−1.1 (−4.5, 2.4)

Serum ferritin, *μ*g/L		Mean^2^ (95% CI)	Mean^2^ (95% CI)	Mean^3^ (95% CI)

Weekly Fe+ Zn	88	40.1 (32.0, 50.4)	52.1 (44.5, 61.1)	29.9% (10.6, 52.6)^4^
Weekly Fe	97	41.2 (33.2, 51.2)	55.5 (47.6, 64.6)	34.6% (15.4, 57.0)^4^
Daily Fe+ Zn	84	39.0 (30.8, 49.3)	74.6 (63.2, 88.0)	91.2% (61.9, 125.9)^5^
Daily Fe	100	41.5 (33.6, 51.4)	75.8 (65.2, 88.2)	82.6% (57.0, 112.4)^5^

Serum zinc, *μ*mol/L		Mean^1^ (95% CI)	Mean^1^ (95% CI)	Mean^3^ (95% CI)

Weekly Fe+ Zn	88	10.0 (9.5, 10.4)	10.4 (9.9, 10.8)	0.4 (−0.2, 1.0)
Weekly Fe	97	10.5 (10.1, 10.9)	10.5 (10.1, 11.0)	0.0 (−0.5, 0.6)
Daily Fe+ Zn	84	10.9 (10.4, 11.3)	10.8 (10.3, 11.2)	−0.1 (−0.7, 0.5)
Daily Fe	100	10.7 (10.3, 11.1)	10.6 (10.2, 11.0)	−0.1 (−0.7, 0.4)

^1^Least square mean (95% CI) from generalized linear model (Proc Mixed).

^2^Geometric mean (95% CI).

^3^Mean percentage (95% CI) difference between endline and baseline values.

^4, 5^Values in column with superscripts without a common number differ significantly, *P* < 0.05.

**Table 3:** Anemia, iron, and zinc deficiency in women before and after daily or weekly supplementation for 12 week.

Treatment groups	Baseline **(Percent)**	Endline **(Percent)**
Anemia (Hb <120 g/L)		
Overall	**48 (13.0)**	**53 (14.5)**
Weekly Fe+ Zn	13 (14.8)	16 (18.4)
Weekly Fe	13 (13.4)	13 (13.7)
Daily Fe+ Zn	13 (15.5)	12 (14.5)
Daily Fe	9 (9.0)	12 (12.0)

Insufficient iron stores (serum ferritin <20 *μ*g/L)		
Overall	**72 (19.7)^1^**	**24 (6.6)^2^**
Weekly Fe+ Zn	20 (22.7)^1^	9 (10.2)^2^
Weekly Fe	21 (21.9)^1^	8 (8.3)^2^
Daily Fe+ Zn	16 (19.3)^1^	4 (4.8)^2^
Daily Fe	15 (15.2)^1^	3 (3.0)^2^

Depleted iron stores (serum ferritin <12 *μ*g/L)		
Overall	**42 (11.5)** ^1^	**10 (2.7)^2^**
Weekly Fe+ Zn	11 (12.5)^1^	5 (5.7)^2^
Weekly Fe	11 (11.5)^1^	1 (1.0)^2^
Daily Fe+ Zn	8 (9.6)^1^	2 (2.4)^2^
Daily Fe	12 (12.1)^1^	2 (2.0)^2^

Zinc deficiency (serum zinc <10.7 *μ*mol/L)		
Overall	**198 (54.4)**	**207 (56.9)**
Weekly Fe+ Zn	56 (63.6)	53 (60.2)
Weekly Fe	51 (53.7)	61 (64.2)
Daily Fe+ Zn	42 (50.6)	44 (53.0)
Daily Fe	49 (50.0)	49 (50.0)

^1,2^Values in column with superscripts without a common number differ significantly, *P* < 0.05.
